# Combined Production of Astaxanthin and β-Carotene in a New Strain of the Microalga *Bracteacoccus aggregatus* BM5/15 (IPPAS C-2045) Cultivated in Photobioreactor

**DOI:** 10.3390/biology10070643

**Published:** 2021-07-10

**Authors:** Konstantin Chekanov, Daniil Litvinov, Tatiana Fedorenko, Olga Chivkunova, Elena Lobakova

**Affiliations:** 1 Department of Bioengineering, Faculty of Biology, Lomonosov Moscow State University, 1-12 Leninskie Gory, 119192 Moscow, Russia; danon6868@gmail.com (D.L.); tatfed@mail.ru (T.F.); olga.chivkunova@mail.ru (O.C.); elena.lobakova@gmail.com (E.L.); 2 Centre for Humanities Research and Technology, National Research Nuclear University MEPhI, 31 Kashirskoye Highway, 115522 Moscow, Russia

**Keywords:** photobioreactors, astaxanthin, β-carotene, carotenoids, carotenogenic algae, *Bracteacoccus*

## Abstract

**Simple Summary:**

Microalgae are the richest source of natural carotenoids, valuable pigments, which are key components of functional food, cosmetics, drugs and animal feeding. To date, only two genera of green microalgae are widely used for mass production of carotenoids on an industrial scale, *Haematococcus* and *Dunaliella*. They produce astaxanthin and β-carotene, respectively, which are among the most useful carotenoids. In doing so one alga produces only one type of a pigment. In this paper we characterize a new strain of the green microalga *Bracteacoccus aggregatus* BM5/15 (IPPAS C-2045). It can simultaneously produce both carotenoids (up to 13.1 and 47.9% β-carotene and astaxanthin of cell dry mass, respectively). Growth parameters of the strain cultivated in glass bubble-column photobioreactors for commercial cultivation of microalgae were obtained. We also provide data of microscopic observations, pigment and fatty acid profile of the microalga, which are important biotechnological characteristics. Collectively, new data makes *B. aggregatus* BM5/15 suitable for industrial production of β-carotene, a pro-vitamin A, and astaxanthin, the most powerful antioxidant.

**Abstract:**

Carotenoids astaxanthin and β-carotene are widely used natural antioxidants. They are key components of functional food, cosmetics, drugs and animal feeding. They hold leader positions on the world carotenoid market. In current work, we characterize the new strain of the green microalga *Bracteacoccus aggregatus* BM5/15 and propose the method of its culturing in a bubble-column photobioreactor for simultaneous production of astaxanthin and β-carotene. Culture was monitored by light microscopy and pigment kinetics. Fatty acid profile was evaluated by tandem gas-chromatography–mass spectrometry. Pigments were obtained by the classical two-stage scheme of autotrophic cultivation. At the first, vegetative, stage biomass accumulation occurred. Maximum specific growth rate and culture productivity at this stage were 100–200 mg∙L^−1^∙day^−1^, and 0.33 day^−1^, respectively. At the second, inductive, stage carotenoid synthesis was promoted. Maximal carotenoid fraction in the biomass was 2.2–2.4%. Based on chromatography data, astaxanthin and β-carotene constituted 48 and 13% of total carotenoid mass, respectively. Possible pathways of astaxanthin synthesis are proposed based on carotenoid composition. Collectively, a new strain *B. aggregatus* BM5/15 is a potential biotechnological source of two natural antioxidants, astaxanthin and β-carotene. The results give the rise for further works on optimization of *B. aggregatus* cultivation on an industrial scale.

## 1. Introduction

Carotenoids are widely used by humans. Due to the high number of healthy properties, they are key components of drugs, cosmetics, biologically active additives and other health food products [1,2,3,4,5,6,7,8]. They are also considered as lipophilic food colorants [3,9]. Carotenoids are powerful natural antioxidants. Particularly, antioxidant activity of astaxanthin is 100 times higher than of vitamin E [6]. Many carotenoids, such as β-carotene, are precursors of vitamin A. Therefore, they are an essential component of human diet [2]. Astaxanthin is not a pro-vitamin A. At the same time, it exhibits anti-inflammatory, antitumor, UV-protective and many other health benefit effects [4,6,10,11,12,13]. Astaxanthin determines the colorful appearance of some animals. Such red colorations of shells of crayfish, lobsters, crabs and shrimps, meat of salmonids, as well as a rose plumage of flamingo are due to the presence of this carotenoid in their diet [14,15,16,17]. It makes astaxanthin an essential component of feed, especially in aquaculture. For the last 10 years astaxanthin and β-carotene have retained leading positions on the world market of carotenoids. According to the Grand View Research council (https://www.grandviewresearch.com/, accessed on 10 April 2021), in 2017, the total carotenoid world market was estimated at USD 2 billion. A gradual increase by 2027 is projected.

In industry, most carotenoids are obtained by chemical synthesis. Costs of the synthetic pigments are lower than natural ones [1,8], but their application has some limitations. Particularly, they cannot be used for medicines, cosmetics and food. Therefore, the task of obtaining carotenoids with the help of metabolic pathways of living things is still necessary.

On an industrial scale, microalgae are cultivated either extensively or intensively. In the first case, the cultures are grown in open ponds occupying large areas [1,18,19,20,21,22,23,24,25]. In outdoor ponds the culture is often illuminated by sunlight. Such systems are characterized by poorly controlled conditions; the culture there is highly vulnerable to contamination. At the same time, operation costs of culturing in open ponds are relatively low. In the second case, cultivation takes place in closed fermenters, photobioreactors, where microalgae can be illuminated by artificial light [8,26,27]. A photobioreactor is an enclosed, illuminated culture vessel designed for controlled biomass production [27]. There, it is possible to provide sterility or stable composition of satellite bacterial communities of an alga. Particularly, *H. lacustris* cultures in photobioreactor are characterized by specific robust bacterial composition different from that of an outside laboratory [28]. Using photobioreactors allows to maintain relatively stable culturing conditions, and therefore culture productivity in photobioreactors is more predictable. The most common strategy for production of natural carotenoids from microalgae is two-phase (or two-stage) cultivation [20,21,29,30,31]. At the first, “vegetative”, stage biomass accumulation occurs. At the second, “inductive”, stage carotenoids accumulation is promoted under stressful conditions. Another approach, one-stage cultivation, is based on reconciliation of vegetative growth and carotenoid accumulation [23,30].

Some microalgae accumulate high amounts of carotenoids. They are considered as an industrial source of natural pigments [1,32,33]. Two species of green microalgae are cultivated on the industrial scale for production of carotenoids: *Dunaliella salina* (formerly *D. bardawil*) (Chlamydomonadales) [19,20,21,31,34] and *Haematococcus lacustris* (formerly *H. pluvialis*) (Chlamydomonadales) [14,35,36,37,38]. Industrial strains of *D. salina* accumulate up to 14% β-carotene of cell dry mass (DM) [1,20,21]. *H. lacustris* is a source of astaxanthin. Under large scale cultivation conditions, it accumulates 2–3% of DM of astaxanthin [1,36,37]. There are some other microalgae considered as potential carotenoid producers, except these two ‘canonical’ species. Particularly, there are pilot studies on assessment of biotechnological potential and carotenoid composition of *Coelastrella* (formerly *Scotiellopsis*) *rubescens* (Sphaeropleales) [39]. The microalga accumulates predominantly astaxanthin and intermediates of its synthesis as well as α/β-carotene under autotrophic growth conditions [40]. In the presence of CH_3_COONa a high fraction of canthaxanthin and pigments of violaxanthin cycle (violaxanthin and zeaxanthin) are observed in the biomass of *C. rubescens* [40]. *Chromochloris zofingiensis* (formerly *Chlorella zofingiensis*) (Sphaeropleales) is one of the most extensively studied carotenogenic microalgae. It accumulates carotenoids, and, like other carotenogenic microalgae, triacylglycerols, under different stress factors, e.g., nitrogen starvation [22,41,42]. *Ch. zofingiensis* accumulates high amounts of lutein at early stages of cultivation, and later three predominant ketocarotenoids—astaxanthin, canthaxanthin and adonixanthin [22,43,44]. Its maximal astaxanthin content is 0.6% of cell DM [42], which is lower than in *H. lacustris*. Astaxanthin (and other ketocarotenoid) yield can be increased under mixotrophic conditions: addition of glucose led to an increase of astaxanthin, canthaxanthin and adonixanthin [45]. However, addition of CH_3_COONa has not resulted in the increase of carotenoid production [44]. There are some microalgae considered as lutein (another widely used carotenoid) producers [46,47,48]. For example, *Muriellopsis* sp. accumulates 150 mg·m^−2^·day^−1^ of the pigment, which is comparable to production of astaxanthin and β-carotene in *Haematococcus* and *Dunaliella*, respectively [46]. Its specific growth rate has been estimated as high as 0.17 day^−1^ [46]. The strain *Scenedesmus* sp. CCALA 1074 isolated from the plankton of the Sihl river (Switzerland) [49] accumulates lutein and mixture of ketocarotenoids. The cyanobacteria *Arthrospira* spp. (formerly *Spirulina* spp.) are also considered as a source of carotenoids. *A. platensis* accumulates up to 15% of carotenoids. Its major carotenoids are β-carotene and zeaxanthin [50].

Isolation and characterization of new microalgae with a putative biotechnological potential is a topical issue. New species of the *Haematococcus* genus, *H. rubicundus* and *H. rubens* were characterized in Central Europe [51,52]. *H. rubicundus* also was isolated in the White Sea coastal rock ponds [53]. A new species, *H. alpinus*, was found in New Zealand [54]. Procházková et al. [55] described a new genus *Sanguina* (Chlamydomonadales, Chlamydomonadaceae) accumulating carotenoids. Carotenogenic microalgae often occupy ecological niches characterized by adverse conditions, e.g., melting snow or temporal rock ponds, due to their high stress tolerance, [32,33,56]. Strains of carotenogenic microalgae from the genera *Haematococcus*, *Coelastrella* and *Bracteacoccus* were isolated previously in the White Sea coastal zone [53,57]. The goal of current work is to describe carotenoid composition and productivity of the new strain of the freshwater green microalga *B. aggregatus* BM5/15 (IPPAS C-2045) from the White Sea region [53] cultivated autotrophically in bubble-column photobioreactors.

## 2. Materials and Methods

### 2.1. Strain and Vegetative Growth Conditions

The strain *Bracteacoccus aggregatus* BM5/15 of the freshwater green microalga (Sphaeropleales, Bracteacoccaceae) has been isolated from the White Sea coastal zone and identified previously [53]. The strain was deposited to the collection of microalgae of Institute of Plant Physiology of Russian Academy of Sciences, under the code IPPAS C-2045. Common two-phase strategy [30] was applied for culturing of the microalga. At the vegetative phase, the cells were grown in 400 mL of the BG-11 mineral medium [58] in 600 mL bubble-column glass photobioreactors (6.6 cm internal diameter, 1.5 L volume) at 20 °C under continuous illumination by cold white light (60 μmol·m^−2^·s^−1^). The light photon flux density was measured by a LiCor 850 quantum meter with a cosine-corrected sensor (LiCor, Lincoln, NE, USA) in the center of an empty column. The culture was bubbled by the air with atmospheric CO_2_ concentration (0.8 L·min^−1^). Initial culture density was of 15 mg·L^−1^ of total chlorophyll content, that corresponded to 0.2 mg·L^−1^ of DW.

Since industrial cultures of carotenogenic microalgae often are not axenic [14,37], we used non-axenic culture of *B. aggregatus* BM5/15. The strain was represented by unialgal culture, i.e., by the culture of one oxygenic photoautotrophic microorganism. At the same time, the culture was represented by a community with bacteria. No contamination by other microalgae and heterotrophic eukaryotes was detected in previous genetic study [53].

### 2.2. Carotenoid Synthesis Induction

The induction of carotenoid accumulation was performed by increasing the light photon flux density to 460 μmol·m^−2^·s^−1^ and transferring the cells either to the sterile distilled water or the NaNO_3_-free BG-11_0_ [59] medium. Previously it was considered as optimal conditions for carotenoid production in the used culturing system [57,60]. The cells of *B. aggregatus* BM5/15 were cultured for 10 days under the inductive conditions.

### 2.3. Dry Mass Determination

Cell DM was determined gravimetrically [61]. The cells from a known volume of suspension were transferred to the GF/F glass fiber filters (Whatman, Dassel, Germany). The filters with the cells were dried to a constant mass in a microwave oven. The mass of filters with dried cells as well as mass of empty dried filters were determined on an analytical balance.

For fitting of the dynamic of DM or pigment content on the vegetative growth stage, the standard Verhulst equation [62,63] was used:

(1) $$DM\left(t\right)=\frac{DM_{max}}{1+\left(DM_{max}/DM_{0}-1\right)e^{-\mu \left(t-L\right)}}$$

where DM(t) is the DM depending on time, *t*, $DM_{max}$, the maximal DM content, $DM_{0}$, the initial DM content, *μ*, the maximum specific growth rate, and *L*, the lag phase duration, are constants. Fitting was performed in Origin (OriginLab Corporation, MA, USA).

### 2.4. Microscopic Observations

In order to assess the state of the culture, possible contaminations and bacterial component of *B. aggregatus* BM5/15, microalgal cell suspensions were evaluated on a Leica DM2500 (LEICA Microsystems, Wetzlar, Germany) light microscope equipped by a photocamera of the same manufacturer during cultivation time. For registering of the motile cells, 0.1% (*v*/*v*) of formaldehyde was added to suspensions to fix them.

### 2.5. Pigment Extraction

Routinely, pigments were extracted with dimethyl sulfoxide as described previously [64]. Chlorophyll *a* and *b* as well as total carotenoid concentrations in obtained extracts were determined spectrophotometrically on an Agilent Cary 300 (Agilent, CA, USA) using the previously published equations [65].

For chromatographic analysis 50–70 mg of cell DM were disrupted at the temperature of liquid nitrogen boiling using a ceramic mortar and a pestle. Then, pigments were extracted according to the standard method by Folch et al. [66]. Chloroform fraction was used for the subsequent analyses.

### 2.6. Chromatographic Analysis

#### 2.6.1. Thin Layer Chromatography

For thin layer chromatography (TLC) chloroform pigment extracts were transferred to silica gel plates Sulifol (Kavalier, Prague, Czech Republic). The system of organic solvents hexane:chloroform:benzene = 10:20:1 (by volume) was used for separation. It was previously developed for extracts of carotenogenic algae [57]. Plates with the pigments were transferred to the atmosphere of saturated vapor of the solvents for pigment separation at room temperature. The retardation indices (*Rf*) were calculated for each pigment fraction using the distances from the starting point to the geometrical center of spots corresponding to each pigment fraction. Pigment fractions obtained by TLC were eluted from silica gel by 100% acetone, hexane or chloroform. Pigments were determined by their *Rf* [53,57] and absorbance spectra [67]. Spectra were registered on an Agilent Cary 300 (Agilent, CA, USA) spectrophotometer in standard 1 cm quartz cuvettes. Pigment contents in the fractions were determined using the extinction coefficients from [67,68].

#### 2.6.2. Gas Chromatography–Mass Spectrometry

To estimate content of different fatty acid (FA) residues in the *B. aggregatus* BM5/15 biomass, tandem gas chromatography–mass spectrometry was applied. Chloroform fractions obtained as described in Section 2.5 were used for the analysis. The chloroform was evaporated in the Ar atmosphere at room temperature, and the lipid residue was dissolved in methanol. FA methyl esters were prepared by transesterification of the lipids by refluxing for 1.5 h in methanol containing 5% concentrated sulfuric acid at 70 °C [69]. Methyl esters were extracted with *n*-hexane and immediately used for gas chromatography. The FA methyl esters were separated and identified according to retention times of standards (Sigma, St. Louis, MO, USA) and by characteristic mass spectra obtained with an Agilent 7890 gas chromatograph equipped with a 30-m HP5MS UI capillary column coupled with an Agilent 5970 mass-selective detector (Agilent, CA, USA). He at a flow rate of 1 mL min^−1^ was used as a carrier gas. Relative FA content was calculated as the mass fraction of their total content in the sample (%-mass). The unsaturation index (UI) [70] was calculated as:

(2) $$UI=\underset{i}{\sum }UFA_{i}/\underset{i}{\sum }SFA_{i}$$

where *UFA_i_* is the mass fraction of the unsaturated FA *i* (mass.%), *SFA_i_* is the mass fraction of the saturated FA *i* (mass.%) in the sample.

### 2.7. Data Treatment

The experiments on *B. aggregatus* BM5/15 culturing were performed in three biological replicates with two analytical replicates for DM and pigment content determination. Average values and their standard deviations are shown.

## 3. Results and Discussion

### 3.1. Evaluation of B. aggregatus BM5/15 Cell Morphology

**Figure 1:**
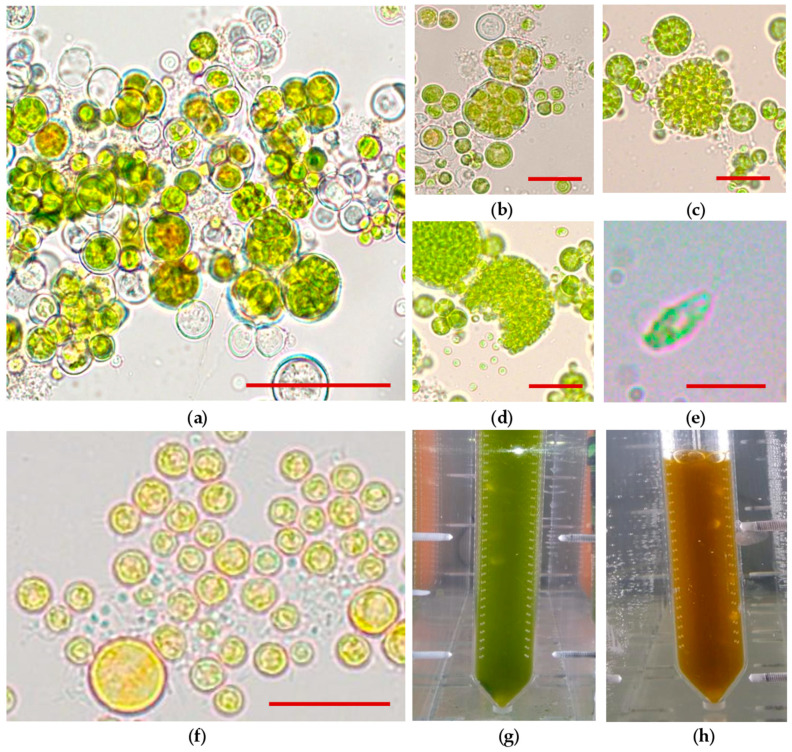
Studies of *Bracteacoccus aggregatus* BM5/15 (IPPAS C-2045) culture by light microscopy and its visual observation in bubble-column photobioreactors. (**a**) Aggregates of palmelloid cells at 2nd day of cultivation under the vegetative growth conditions, scale bar: 20 μm; (**b**) autosporangium under the vegetative growth conditions (5th day), scale bar: 20 μm; (**c**) zoosporangium under the vegetative growth conditions (5th day), scale bar: 20 μm; (**d**) releasing of zoospores under the vegetative growth conditions (5th day), scale bar: 20 μm; (**e**) biflagellate zoospore, scale bar: 4 μm; (**f**) immotile palmelloid cells under the inductive growth conditions (3rd day), scale bar: 10 μm; (**g**) cell suspension under the vegetative growth conditions; (**h**) cell suspension under the inductive conditions.

Cell morphology is an important diagnostic feature of microalgal cultures, especially carotenogenic species, because it reflects metabolic status and division rate of cells [24,39,40]. It is also useful to detect culture contamination. At the vegetative growth stage, *B. aggregatus* BM5/15 cell suspension was green. The culture was presented predominantly by small spherical palmelloid cells (so-called vegetative cells) 5–10 μm diameter (Figure 1a–c). Rarely, large cells up to 25 μm diameter were observed. It was in accordance with morphological features of *B. aggregatus* BM5/15 after its isolation [53]. In the first 1–4 days of cultivation under vegetative growth conditions cells formed large clusters (Figure 1a). Later, the clusters became smaller, and individual cells were predominant. At 5–6 days of cultivation auto- (Figure 1b) and zoosporangia (Figure 1c) appeared in the culture. This period was also characterized by a massive release of motile zoospores (Figure 1d). Zoospores were small (2.5–3.5 μm length) with two flagella on the apex (Figure 1e). To the best of our knowledge, there are no published photographs of *Bracteacoccus* zoospores, but the cells in the *B. aggregatus* BM5/15 culture (Figure 1e) were very similar to drawn illustrations of motile stages of this microalga from Fuíková et al. [71]. High number of zoospores and dividing cells is a sign of actively growing cultures of green carotenogenic algae [60,72,73,74]. Thus, their mass appearance in *B. aggregatus* BM5/15 cultures might reflect the high potential of their vegetative growth. At the same time, non-motile vegetative cells are more resistant to adverse conditions [73]. Therefore, older cultures with low number of zoospores (10–12 days old) were better for induction of carotenoid accumulation. Carotenoid synthesis induction by distilled water led to massive cell death and total lysis of the culture at 2nd day of cultivation (data not shown). This method was previously successfully used for induction of astaxanthin synthesis in *H. lacustris* bubbled by air [57]. However, it caused cell lysis in *H. lacustris* cultures bubbled with air–gas mixture enriched with 5% (by volume) CO_2_ [60]. Thus, the transferring of *B. aggregatus* BM5/15 cells to the BG-11_0_ [60] medium was applied for induction of carotenoid accumulation in further experiments even in the case of bubbling with air. No cell division was observed under the inductive conditions, only spherical non-motile cells were in the culture. The cells became yellow (Figure 1f). Visually, carotenoid accumulation in the *B. aggregatus* BM5/15 cultures cultivated in bubble-column photobioreactors was observed as changing the color of cell suspensions from green (Figure 1g) to orange (Figure 1h).

Initially, *B. aggregatus* BM5/15 was isolated as a bacteria-containing unialgal culture (i.e., containing only one strain of oxygenic phototroph, but not free of bacteria) [53]. Single bacteria attached to the surface of algal cells or freely dispersed in the medium at the vegetative and inductive stages of the cultivation were also observed for the strain cultivated in photobioreactors. Often, in laboratory cultures [28,29,75] and natural habitats [75,76] carotenogenic algae exist in a form of complex bacterial communities. Particularly, the carotenogenic chlorophyte *H. lacustris* forms microbial consortia with *Alcaligenaceae*, *Flavobacteriaceae*, *Sphingomonadaceae*, *Comamonadaceae*, and *Caulobacteriaceae* [28,75,76]. Many of them are related to plant growth promoting bacteria, which produce vitamins and other important compounds [77,78]. In some studies, a positive effect of bacteria on *H. lacustris* growth was established [76,77]. At the same time, the protocols for obtaining axenic *H. lacustris* cultures have been developed [76,77,79,80]. The study of *B. aggregatus* bacterial community would be a matter of further research. At the same time, based on microscopic observation, the amount of bacterial biomass is lower than that of microalgae, thus it could not contribute significantly to DM of *B. aggregatus* BM5/15 and its biomass biochemical composition.

### 3.2. Parameters of B. aggregatus BM5/15 Cultured in Photobioreactor

**Figure 2:**
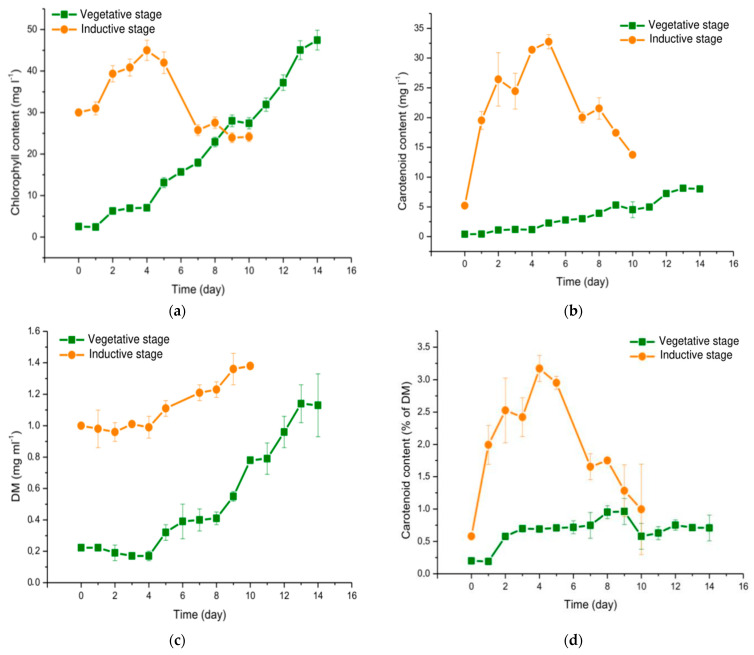
Growth parameters of the *Bracteacoccus aggregatus* BM5/15 (IPPAS C-2045) culture. Green curves, squares (■), correspond to vegetative growth stage. Orange curves, circles (●), correspond to the inductive stage. (**a**) Dynamics of total chlorophyll content in the culture; (**b**) dynamics of total carotenoid content in the culture; (**c**) dynamic of cell dry weight in the suspension; (**d**) dynamics of carotenoid content in the algal biomass. The average values from three biological replicates and standard deviations are shown.

Chlorophyll (Figure 2a) and carotenoid (Figure 2b) contents were gradually increasing in the *B. aggregatus* BM5/15 under the vegetative growth conditions. After 14 days of cultivation, the content of total chlorophyll and carotenoid increased from 2.5 ± 0.0 to 47.5 ± 0.2 mg∙L^−1^ and from 0.4 ± 0.0 to 8.1 ± 0.1 mg∙L^−1^, respectively. DM increased at the vegetative growth stage from 0.2 ± 0.0 to 1.4 ± 0.2 mg∙mL^−1^ (Figure 2c). In previous studies on *B. bullatus* growth [81], maximal DM referred to the same period of time was as high as 2.4 mg∙mL^−1^, which was larger than in the current work. Thus, optimization of the vegetative growth conditions (e.g., increasing initial culture density, changing of culturing medium and light intensity, CO_2_ enrichment) should be the matter of further research. Calculated values of the growth curve parameters by DM accumulation were as follows: *μ* = 0.33 day^−1^, *DM_msx_* = 1.3 mg∙mL^−1^, *L* = 7 days (Table 1). The values of *μ* and *DM_max_* were comparable with the data on the kinetics of carotenogenic algae autotrophic growth. Particularly, *H. lacustris* cultures are characterized by following maximal specific growth rates: *μ* = 0.32 day^−1^ [82], *μ* = 0.31 day^−1^ [83], *μ* = 0.94 day^−1^ (by cell number) [72], *μ* = 0.2 day^−1^ (by cell number) [84]. For *H. alpinus* it is 0.31 day^−1^ [63]. *C. rubsecens* is characterized by *μ* = 0.35 day^−1^ (by cell number) [39]. For *Ch. zofingiensis* it is estimated as 0.77 day^−1^ [44], as 0.5 day^−1^ (by cell number) [85], as 0.96 day^−1^ [22], or as 0.72 day^−1^ (by cell number) [22]. In lutein-producing *Scenedesmus* sp. CCALA 1074, cultivated in a thin-layer photobioreactor, *μ* is of 0.74 day^−1^ [49]. Thus, the value of *μ* of *B. aggregatus* BM5/15 culture is comparable with that reported for other main carotenogenic microalgae. At the same time, the lag phase was too long. Therefore, increasing biomass yield would be achieved by increasing the initial culture density and performing multiple reseeding of the culture before growth in photobioreactors in order to obtain the population of actively dividing cells. Total carotenoid content in the algal biomass did not change significantly under the conditions of vegetative growth and was in the range of 0.2–1.0% of DM (Figure 2d).

Under the inductive conditions, chlorophyll content was gradually increasing during the first four days of cultivation up to 52.1 ± 2.8 mg∙L^−1^. Then, it decreased sharply (Figure 2a). Increasing chlorophyll content at the induction stage of cultivation is not a typical feature of carotenogenic algae. In *H. lacustris*, it decreased monotonously after transferring to new conditions [38,60,86,87]. The same is true about *Ch. zofingiensis* cultures [41,45]. Chlorophylls are directly involved in photosynthesis. Decrease of their content under inductive conditions is accompanied by downregulation of photosynthetic activity [60,87,88,89,90]. Thus, chlorophyll increase in the *B. aggregatus* BM5/15 culture might be considered as the sign of retention of photosynthetic activity under stress. Cell DM also increased in *B. aggregatus* BM5/15 cultures under the inductive conditions: it raised from 1.0 ± 0.0 to 1.4 ± 0.0 mg∙L^−1^ (Figure 2c). There were no signs of cell division at the inductive stage. At the same time, biomass increasing is explainable for microalgae, because carotenoid synthesis under inductive conditions is accompanied by massive triacylglycerol accumulations [41,44,91,92,93]. Carotenoid content in the algal biomass was sharply increasing during the first five days at the inductive stage. It reached 25.8 ± 1.3 mg∙L^−1^ (Figure 2b) and 2.3 ± 0.1% of cell DM on the 5th day (Figure 2d). In *H. lacustris* and *D. salina* carotenoid contents reach 3–5% [14,30,35,36,37] and 14% [1,19,20,21,31,34], respectively. However, other microalgae demonstrate more modest carotenoid yields. In *C. rubescens* carotenoid content reaches 2% [39,40]. Maximal reported carotenoid content in *Ch. zofingiensis* is of 1% [22,44,45]. *Scenedesmus* sp. CCALA 1074 accumulates up to 2.3% of DM of carotenoids [49].

After the 5th day of cultivation at the inductive stage carotenoid content in *B. aggregatus* BM5/15 biomass decreased (Figure 2b,d). It might be due to pigment destruction and/or ongoing accumulation of triacylglycerols. A similar situation was observed previously in other strains of carotenoid-producing algae [22,35,57,88].

Collectively, *B. aggregatus* BM5/15 cultivated in the bubble-column photobioreactors was characterized by an average productivity of 100–200 mg∙L^−1^∙day^−1^, and carotenoid yield of 3.6–3.8 mg∙L^−1^∙day^−1^. The data on main culture parameters are summarized in Table 1.

### 3.3. Carotenoid Profile in the B. aggregatus BM5/15 Biomass at the Induction Stage

In the cells of microalgae, primary and secondary carotenoids are distinguished. In the first case, pigments are coupled with photosynthetic apparatus. Their amount is limited by and under strong control of the cell. In the second case, carotenoids are synthesized independently on photosynthetic apparatus. Therefore, pigment level is not limited by cell photosynthetic capacity, and their content reaches high yield [32,36,94]. Under the inductive conditions, pigment content in the *B. aggregatus* BM5/15 cells sharply raised (Figure 2b,d). The culture demonstrated signs of secondary carotenoids accumulation (see above).

**Figure 3:**
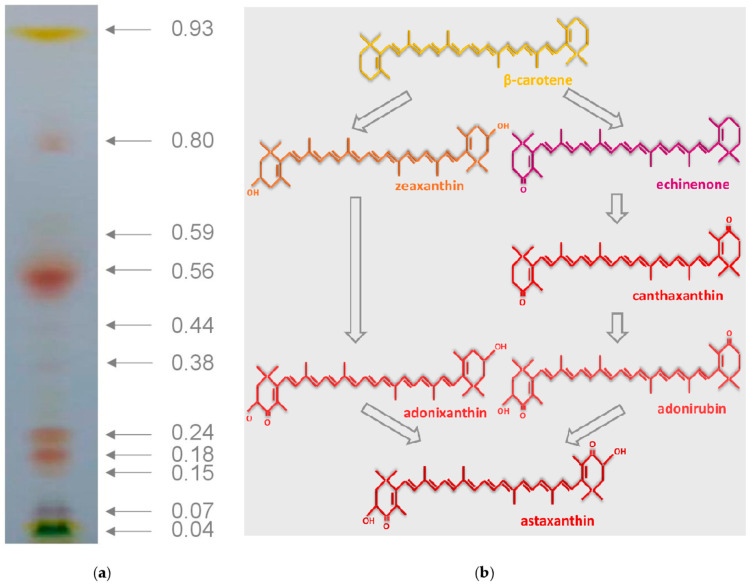
Carotenoid composition of *Bracteacoccus aggregatus* BM5/15 (IPPAS C-2045) at the inductive stage of cultivation (5th day). (**a**) Representation results of separation of chloroform pigment extracts from the *B. aggregatus* BM5/15 cells after five days of cultivation under the inductive conditions. Values of the retardation factor are presented for each pigment fraction. (**b**) Putative pathways of xanthophyll synthesis in *B. aggregatus* BM5/15 based on its pigment profile.

A total of 11 pigment fractions were obtained from the extracts of *B. aggregatus* BM5/15 cultivated under the inductive conditions for five days (Figure 3a). Based on the *R_f_* and absorption spectra [67] the following pigments were observed in the total extract: β-carotene, adonirubin, adonixanthin, echinenone, canthaxanthin, astaxanthin, primary xanthophylls (violaxanthin, zeaxanthin, antheraxanthin, neoxanthin, and lutein), pheophytins, and chlorophylls (Table 2). Astaxanthin, adonixanthin and β-carotene were predominant pigments in the cells, their fractions were ca. 48, 23 and 13%, respectively. Astaxanthin, adonixanthin and adonirubin, possessing hydroxyl group(s), might be deposited in the cells of *B. aggregatus* BM5/15 mainly in the form of FA esters. Formation of esters with FA makes it possible to accumulate carotenoids in the hydrophobic environment of lipid globules, which play the role of a cell compartment for storage of lipophilic molecules [91,95,96]. Diesters represented the majority of astaxanthin forms in *B. aggregatus* BM5/15. It is in accordance with previous data on the pigment profile of *B. minor* under inductive conditions [70]. In primary work on *B. aggregatus* BM5/15 with non-optimized method of carotenoid synthesis induction [53], similar results had been obtained. Astaxanthin and β-carotene were also predominant, but β-carotene fraction was higher and most of astaxanthin was in the form of monoesters. Thus, it would be possible to modulate carotenoid composition of *B. aggregatus* BM5/15 by variation of the induction method.

In chemical terms, carotenoids are representatives of terpenoids, molecules of biological origin, of which the C5 hydrocarbon isoprene is a structural unit. They are related to tetraterpenes (C40-isoprenoids), i.e., they consist of eight isoprene building blocks [67]. In cells of higher plants and most of algae they take their origin in plastids, where their precursor, isopentenyl pyrophosphate (or ‘activated isoprene’), is synthesized via 1-deoxy-*D*-xylulose-5-phosphate pathway [2,32,94]. However, there are some exceptions. Particularly, euglenophytes synthesize carotenoids via mevalonate pathway and cyanobacteria realize all stages of their synthesis in cytosol [32]. C-5 precursors are condensed into the C-40 terpenoid phytoene and then desaturated to the non-cyclic lycopene. After desaturation, α and/or β-ionone are formed on the ends of carotenoid molecules [2,32,94]. Carotenoids are divided into oxygen-free carotenes and oxygen-containing xanthophylls [94]. Astaxanthin is produced from β-carotene by addition of two keto- and two hydroxyl groups with the help of corresponding enzymes (Figure 3b). Some authors proposed a particular chain of reactions in different carotenogenic microalgae. According to Han et al. [95], in *H. lacustris*, ketogroups are added initially, thus astaxanthin is formed through echinenone, canthaxanthin and adonirubin, whereas in *Ch. zofingiensis* hydroxyl groups are formed first leading to formation of the adonirubin intermediate. In other works, all types of reactions are principally possible in one microalga [43,44,72]. Based on results of our work the following metabolic nexus of astaxanthin biosynthesis was possible in *B. aggregatus* BM5/15 (Figure 3b). Flux of β-carotene precursors was being implemented through both two pathways: with either primary ketolation or hydroxylation of ionone rings. In the first case, the pigment was formed through echinenone, canthaxanthin and adonirubin. In the second case, zeaxanthin and adonirubin were astaxanthin synthesis intermediates. Principally, the formation of adonixanthin from echinenone and adonirubin from cryptoxanthin were also possible, but corresponding intermediates, 3-hydroxyechinenone and 3′-hydroxyechinenone, were not detected in *B. aggregatus* BM5/15.

Pigment composition has been evaluated for some carotenogenic microalgae after carotenoid synthesis induction. In *H. lacustris*, astaxanthin is a single predominant carotenoid, only a minor fraction of other pigments is presented [57,72,97]. In other species the fraction and diversity of intermediates are higher [39,40,42,43,44,45]. The strains defined as *Neochloris wimmeri* CCAP-213/4, *Coelastrella oocystiformis* SAG-277/1, and *Protosiphon botryoides* SAG731/1a accumulate significant amounts of astaxanthin monoesters and minor fraction of other ketocarotenoids [97]. The strain *Scenedesmus vacuolatus* SAG-211/15 accumulates predominantly canthaxanthin and lutein [97]. The microalga *Bracteacoccus minor* accumulates a mixture of ketocarotenoids: astaxanthin, canthaxanthin, echinenone, adonixanthin and adonirubin [24,70]. Astaxanthin diesters are its predominant form [24,70]. High astaxanthin yield in *H. lacustris* may be explained by effective channeling of substrates due to high degree of interaction between enzymes of xanthophyll synthesis [98]. At the same time, less intense channeling and enzymes activity make it possible to obtain valuable intermediates, e.g., β-carotene. Most likely, the last situation was possible in *B. aggregatus* BM5/15. Thus, the microalga may be considered as a source of both carotenoids, astaxanthin and β-carotene.

### 3.4. Fatty Acid Profile B. aggregatus BM5/15 Biomass at the Induction Stage of Cultivation

In *B. aggregatus* BM5/15 cells from both, vegetative and inductive, cultivation stages palmitic (C16:0), oleic (C18:1^Δ9^) and linoleic (C18:2^Δ9,12^) FA residues were predominant (Table 3). It is in accordance with the data on FA composition of most green algae and higher plants [39,40,43,50,99,100,101] At the inductive stage, α-linolenic acid (C18:3^Δ9,12,15^) residues also became predominant.

As a rule, accumulation of secondary carotenoids in algae is accompanied by an increase of neutral lipid (especially triacylglycerols) content. For example, in *H. lacustris*, FA residues content correlates with astaxanthin content [91]. Thus, carotenogenic microalgae are often considered as a source of biofuel [102,103]. Previously *Bracteacoccus* was considered as triacylglycerols producers. The strains of *B. bullatus* were proposed as a potential source of biodiesel [81,104]. This species is also characterized by predominance of palmitic, oleic and linoleic FA residues. Under stress conditions, the strain *B. bullatus* MZ-Ch11 also accumulates α-linolenic acid, but its content in the lipid fraction was significantly lower than in *B. aggregatus* BM5/15 (lower than 0.3%) [81]. Another strain, *B. bullatus* MZ-Ch32, accumulates up to 21.4% of this FA [104]. FA composition of carotenoid ester fractions was similar to that of whole microalgal neutral lipid extracts [57]. Most likely, in *B. aggregatus* BM5/15, carotenoid ester FA composition also was comparable to total FA profile.

Accumulation of *B. aggregatus* BM5/15 accompanied by an increase of the UI (Table 3). By contrast, in the microalga *C. rubescens* the content of α-linolenic acid residues and UI decreased during carotenoid accumulation [40]. The same is true about *Ch. zofingiensis*. In its cells UI also decreased during carotenogenesis. The content of α-linolenic acid in this alga is low in both cases: vegetative growth and carotenogenesis induction [43,44]. In *D. salina* carotenogenesis is also accompanied to decrease of this FA [92].

## 4. Conclusions

We characterized a new strain of *B. aggregatus* BM5/15 (IPPAS C-2045) and proposed a technology of its culturing in glass bubble-column photobioreactor for carotenoid production. Despite the carotenoid content in the *B. aggregatus* BM5/15 biomass is lower than in “champions” of carotenoid accumulation, i.e., *H. lacustris* and *D. salina*, it is higher than in other phototrophically cultivated green carotenogenic algae. At the same time, maximal specific growth rate is higher than in *H. lacustris*. It makes *B. aggregatus* BM5/15 suitable for industrial pigment production. The main new opportunity of the proposed technology is simultaneous production of two widely used carotenoids, a pro-vitamin A β-carotene and the most powerful natural antioxidant astaxanthin.

## 5. Patents

Russian Federation patent #2737139, 11.25.2020.

## Figures and Tables

**Table 1:** Main parameters of the *Bracteacoccus aggregatus* BM5/15 (IPPAS C-2045) growth and carotenoid accumulation in the bubble-column photobioreactors.

Culture Parameter	Value
Maximum specific growth rate (*μ*), day^−1^	0.33
Maximum biomass at the vegetative stage (*DM_max_*), mg∙L^−1^	1.2–1.6
Maximal carotenoid content at the inductive stage, mg∙L^−1^	24.5–27.1
Maximal carotenoid content at the inductive stage, % of dry biomass	2.2–2.4
Average productivity, mg∙L^−1^∙day^−1^	100–200
Carotenoid yield, mg∙L^−1^∙day^−1^	3.6–3.8

**Table 2:** Fractions of carotenoids obtained after separation of the extracts from the cells of *Bracteacoccus aggregatus* BM5/15 (IPPAS C-2045) cultivated for five days under the inductive conditions by thin layer chromatography. Values of the retardation factor (*R_f_*), pigment name based on its *R_f_* value and absorbance spectrum, as well as its mass fraction in the total extract are provided.

*R_f_*	Pigment	Carotenoid Content (Mas.-% of Other Carotenoids)
0.93	β-carotene	13.1
0.80	Adonixanthin diesters	2.4
0.59	Echinenone	1.2
0.56	Astaxanthin diesters	31.1
0.44	Adonirubin esters	8.0
0.38	Canthaxanthin	2.1
0.24	Astaxanthin monoesters	16.8
0.18	Adonirubin monoesters	20.5
0.15	Free ketocarotenoids ^1^	1.4
0.04	Primary xanthophylls ^2^	3.4

^1^ Free astaxanthin and adonixanthin and, possibly, products of their oxidation, ^2^ Xanthophyll associated with photosynthetic apparatus: zeaxanthin, antheraxanthin, violaxanthin, neoxanthin and lutein.

**Table 3:** Fatty acid profile of *Bracteacoccus aggregatus* BM5/15 biomass and calculated values of the unsaturation index (UI).

FA, Mas.-%	Vegetative Stage	Inductive Stage
C12:0	0.1	0.1
C14:0	2.4	1.5
C14:1^Δ7^	0.2	-
C16:0	24.8	26.4
C16:1^Δ7^	3.7	2.4
C16:1^Δ9^	1.6	1.9
C16:2^Δ7,10^	7.3	2.1
C16:3^Δ7,10,13^	0.9	1.9
C16:4^Δ4,7,10,13^	1.2	1.9
C18:0	4.8	2.0
C18:1^Δ9^	17.0	18.8
C18:1^Δ11^	5.0	4.6
C18:2^Δ9,12^	20.4	18.2
C18:3^Δ6,9,12^	0.3	0.2
C18:3^Δ9,12,15^	8.5	17.3
C18:4^Δ6,9,12,15^	0.4	0.4
C20:0	0.7	0.2
C20:1^Δ11^	0.2	0.1
C22:0	0.6	0.2
UI	1.184	1.354

## Data Availability

Not applicable.
